# Meta-analysis of the likelihood of FOXC1 expression in early- and late-stage tumors

**DOI:** 10.18632/oncotarget.26358

**Published:** 2018-11-27

**Authors:** Tsutomu Kume, Tarek Shackour

**Affiliations:** ^1^ Feinberg Cardiovascular and Renal Research Institute, Northwestern University School of Medicine, Chicago 60611, IL, USA

**Keywords:** FOXC1, cancer, T-Stage

## Abstract

**Background:**

Aberrations in the expression of the transcription factor forkhead box C1 (FOXC1) have been linked to a number of malignancies. Here, we characterized the relationship between FOXC1 and cancer progression by conducting a meta-analysis of studies that reported the frequency of FOXC1 expression in tumors of different stages (T1, T2, T3, T4).

**Materials and Method:**

Relevant articles were retrieved from the Medline database by searching for the terms “FOXC1” and “cancer”; then, the retrieved articles were reviewed individually, and studies that were of multivariate cohort design, evaluated FOXC1 expression via immunohistochemical staining, and assessed the relationship between FOXC1 expression and cancer T-stage were included in our meta-analysis.

**Results:**

Our search terms identified 128 studies, 5 of which met all inclusion criteria. A total of 850 tumor samples were evaluated in the 5 studies; 452 samples were from early-stage (T1-T2) tumors, and 398 were from late-stage (T3-T4) tumors. FOXC1 was expressed in 60.7% (516/850) of all samples, in 54.6% (247/452) of early-stage tumor samples, and in 67.5% (269/398) of late-stage tumor samples. When calculated relative to early-stage samples, the pooled risk for FOXC1 expression in late-stage samples was 1.238 (95% CI = 1.061–1.444, *p* = 0.007).

**Conclusions:**

The results from our meta-analysis of 5 studies indicate that FOXC1 is 23.8% more likely to be expressed in late-stage tumors than in early-stage tumors.

## INTRODUCTION

Cancer can arise via the accumulation of single or multiple genetic mutations, which cause the cancer cells to proliferate without restriction: however, the molecular and genetic cascades involved in tumor formation and cancer progression are largely unknown. The forkhead box (FOX) family of transcription factors includes 17 subfamilies, from FOXA to FOXR, that control a wide range of biological processes such as cell growth, proliferation, differentiation, and longevity [1].

The FOXC1 gene encodes a transcription factor that is crucial to mesodermal [2], neural crest [3, 4] and ocular [5–7] development. Heterozygous FOXC1 mutation and copy number variation are associated with Axenfeld-Rieger Syndrome (ARS), which is characterized by anterior eye segment defects, glaucoma, and cerebral small vessel disease (OMIM 601090). In recent years, rapidly accumulating evidence implicates the role of FOXC1 in cancer. FOXC1 is expressed not only in breast cancer subtypes such as basal-like breast cancer (BLBC), but also in hepatocellular carcinoma (HCC), endometrial cancer, Hodgkin’s lymphoma (HL), and non-Hodgkin’s lymphoma (NHL) [8–12]. Increased FOXC1 expression now appears to be linked to more aggressive cancer phenotypes in BLBC, HCC, HL, and NHL [8–12].

The goal of this study was to characterize the relationship between FOXC1 expression and cancer progression by conducting a meta-analysis of studies that reported the frequency of FOXC1 expression in tumors of different stages (T1, T2, T3, T4) [13], and then calculating the pooled relative risk of FOXC1 expression in stage T1-T2 (early) and in stage T3-T4 (late) tumors. We identified 5 reports that met all inclusion criteria and evaluated a total of 850 samples from a wide range of cancer types. Our results suggest that the frequency of FOXC1 expression is significantly higher in late-stage than in early-stage tumors.

## MATERIALS AND METHODS

Our meta-analysis was conducted and reported according to PRISMA for Network Meta-Analyses (PRISMA-NMA) checklist [14]. We used a single database (Medline), which is consistent with the PRISMA-NMA requirements. Relevant studies were retrieved by using the PubMed interface to search for the terms “FOXC1” and “cancer,” and the studies included in our meta-analysis were of multivariate cohort design, evaluated FOXC1 expression via immunohistochemical staining, and assessed the relationship between FOXC1 expression and cancer T-stage (T1, T2, T3, T4). The meta-analysis was performed with an open-source program as described previously [15–18].

## RESULTS

### Study selection

**Figure 1:**
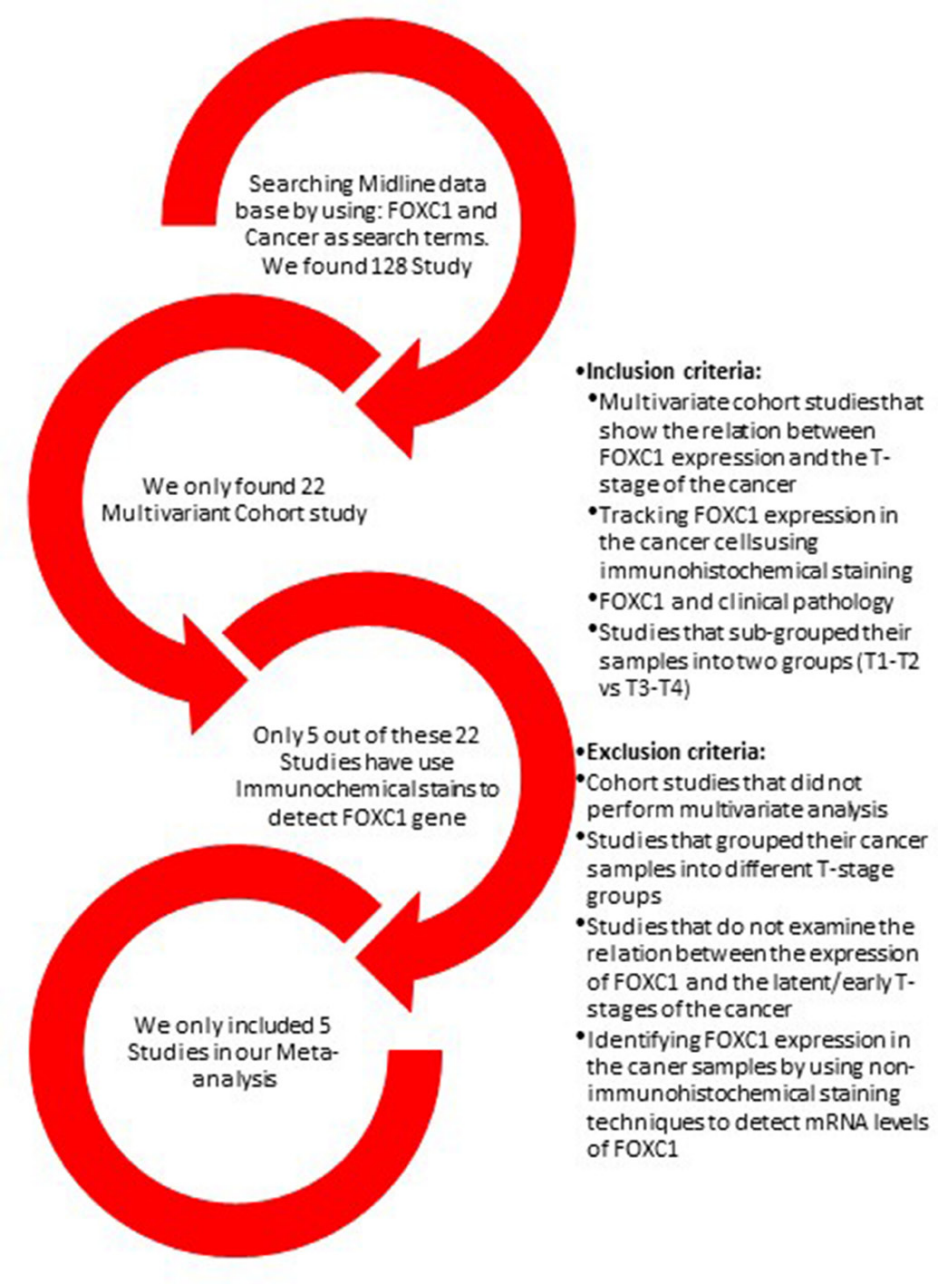
Summary of study selection The Medline database was searched for the terms FOXC1 and Cancer. The studies included in our meta-analysis were multivariate cohort investigations of the relationship between FOXC1 expression and cancer T-stage in which the results were grouped according to tumor stage (T1, T2, T3, T4) and FOXC1 expression was detected via immunohistochemical staining.

The initial Medline search was conducted on September 1st, 2018 and identified 128 articles (Supplementary Table 1); 106 of the studies were excluded because they were not a multivariant cohort studies. Seventeen of the 22 multivariant studies were excluded either because they did not investigate the relationship between FOXC1 expression and cancer, did not categorize their results by tumor stage, or did not evaluate FOXC1 expression immunohistochemically (Figure 1). The remaining 5 studies (Table 1) were multivariate analyses and met all inclusion criteria.

**Table 1:** Summary of studies

Author name	Country	Year	Type of cancer	T1-T2 Tumor sample			T3-T4 tumor sample		
				Total	FOXC1^−^	FOXC1^+^	Total	FOXC1^−^	FOXC1^+^
***Wang, et al.***	China	2017	Salivary Adenoid Cystic Adenocarcinoma	48	24	24	62	29	33
***Xu, et al.***	China	2014	Gastric Carcinoma	34	15	19	86	20	66
***Xia, et al.***	China	2013	Hepatocellular Carcinoma	234	98	136	172	51	121
***Wei, et al.***	China	2013	Non-small cell Lung cancer	61	35	26	64	21	43
***Ray, et al.***	USA	2011	Breast Cancer	75	33	42	14	8	6

### Risk of bias in individual studies

Each individual study had a potential selection bias as the samples for each study were not randomly selected. Furthermore, the immunohistochemical methods used to detect FOXC1 expression varied across the studies, and this variation could also induce bias.

### Data collection

#### Wang, *et al.* (2017) [19]

**Figure 2:**
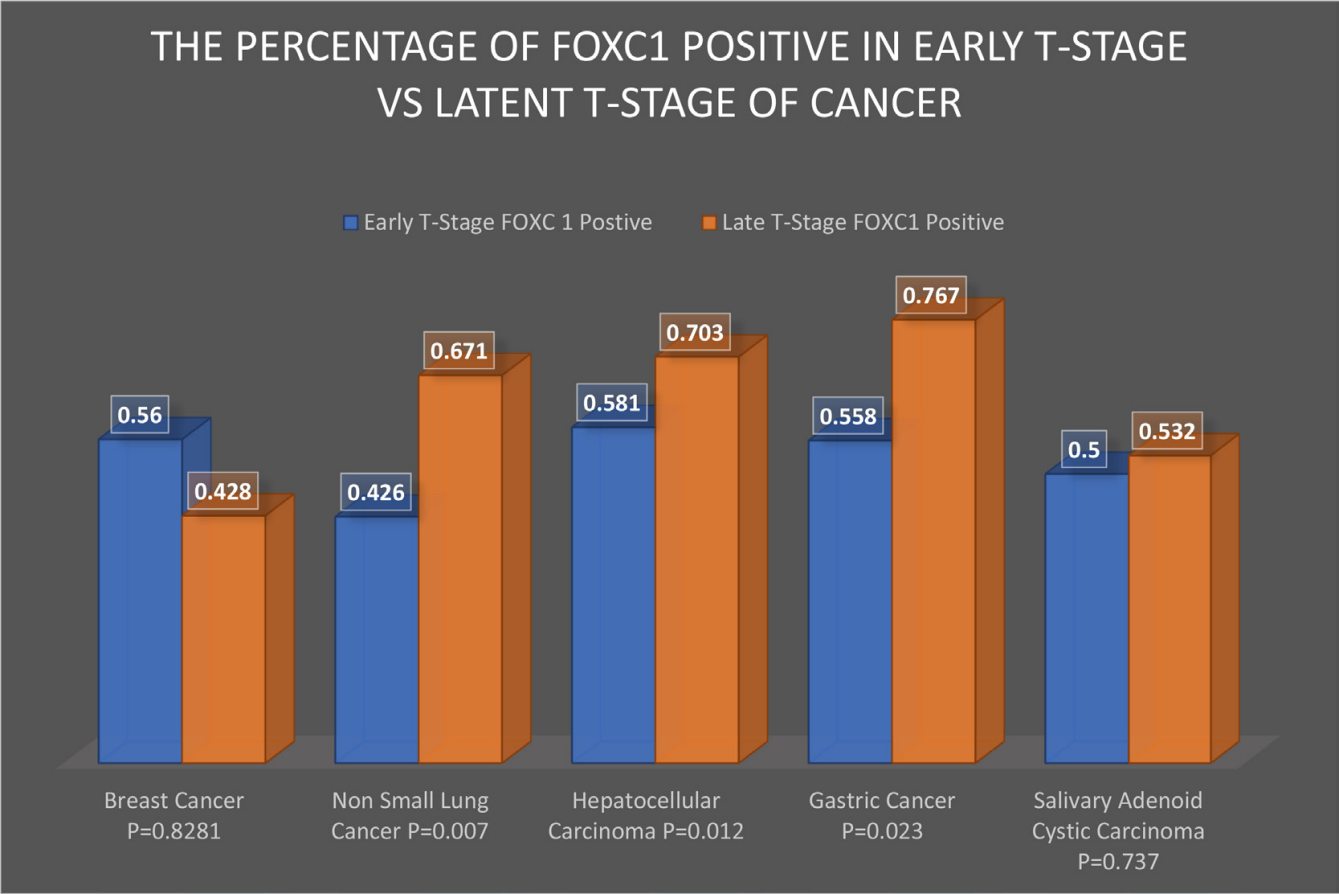
Frequency of FOXC1 expression in early- and late-stage tumors The percentage of early-stage (T1-T2) and late-stage (T3-T4) tumor samples that expressed FOXC1 was calculated for each of the 5 studies included in the meta-analysis and reported according to cancer type.

The authors evaluated 121 tumor samples from patients with salivary adenoid cystic carcinoma; 48 samples were categorized as stage T1-T2 and 62 samples were categorized as stage T3-T4. The proportion of FOXC1-positive tumors in each group was 50% (24/48) and 53% (33/62), respectively (*p*-value = 0.737) (Figure 2). For our meta-analysis, “high” expression levels were considered positive for FOXC1 and “low” expression levels for FOXC1 were considered negative.

#### Xu, *et al.* (2014) [20]

Samples (*n* = 120) were evaluated from the tumors of patients with gastric cancer; 34 samples were from stage T1-T2, 86 were from stage T3-T4, and the proportion of FOXC1 positive tumors in each group was 55.8% (19/34) and 76.7% (66/86), respectively (Figure 2). The authors found that FOXC1 expression was significantly higher in the late T-stage (T3-T4) of the cancer than that in the early T-stage (T1-T2) of the cancer (*p*-value = 0.023).

#### Xia, *et al.* (2013) [21]

Samples (*n* = 406) were evaluated from the tumors of patients with hepatocellular carcinoma. Samples of hepatocellular cancer (>5 cm) are considered stage T3-T4 tumors, and samples (<5 cm) are considered stage T1-T2 tumors [22]; 234 samples were stage T1-T2 tumors,172 samples were stage T3-T4 tumors, and the proportion of FOXC1-positive tumors in each group was 58.1% (136/234) and 70.3% (121/172), respectively (Figure 2). The authors found that FOXC1 expression was significantly higher in the late T-stage (T3-T4) of the cancer than that in the early T-stage (T1-T2) of the cancer (*p*-value = 0.012).

#### Wei, *et al.* (2013) [23]

Samples (*n* = 125) were evaluated from the tumors of patients with non-small lung cancer; 61 samples were from stage T1-T2, 64 samples were from stage T3-T4, and the proportion of FOXC1-positive tumors in each group was 42.6% (26/61) and 67.1% (43/64), respectively (Figure 2). The authors found a significant difference in FOXC1 expression between the two groups. They found a significant increase in FOXC1 expression in the late T-stage (T3-T4) compared to the early T-stage (T1-T2) (*p*-value = 0.007).

#### Ray, *et al.* (2011) [24]

Samples (*n* = 89) were evaluated from the tumors of patients with breast cancer. Samples of breast cancer (>5 cm) are considered stage T3-T4 tumors, and samples (<5 cm) are considered stage T1-T2 tumors [25]; 75 samples were from stage T1-T2, 14 samples were from stage T3-T4, and the proportion of FOXC1-positive tumors in each group was 56% (42/75) and 42.8% (6/14), respectively (Figure 2). The authors found that FOXC1 expression was higher in the early T-stage in compare to the late T-stage. The difference was not significant (*p*-value = 0.8281).

### Synthesis of results

**Figure 3:**
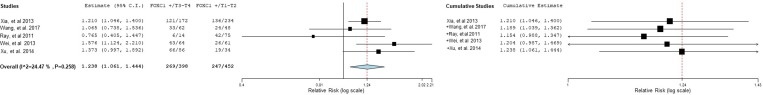
Relative risk of FOXC1 expression in early- and late-stage tumors (individual studies) The relative risk and 95% confidence intervals of FOXC1 expression in early-stage (T1-T2) versus late-stage (T3-T4) and in late-stage versus early-stage tumor samples was calculated for each individual study and displayed in a forest plot.

A total of 850 tumor samples were evaluated in the 5 studies; 452 samples were from stage T1-T2 tumors, and 398 samples were from stage T3-T4 tumors. FOXC1 was expressed in 60.7% (516/850) of all samples, in 54.6% (247/452) of early-stage (T1-T2) tumor samples, and in 67.5% (269/398) of late-stage (T3-T4) tumor samples. Across all five studies, the pooled relative risk for FOXC1 expression in late-stage samples was 1.238 (95% CI = 1.061–1.444, *p* = 0.007) (Figure 3). Thus, the results from our meta-analysis indicate that FOXC1 is 23.8% more likely to be expressed in stage T3-T4 tumors than in stage T1-T2 tumors (Table 2).

**Table 2:** Calculation of pooled relative risk of FOXC1 expression in early- and late-stage tumors (meta-analysis)

Study first author name	Study weight	Pooled relative risk, T3-T4 versus T1-T2				
		Estimate	95% CI lower bound	95% CI upper bound	Standard error	*p*-value
Xia, *et al.*	46.027%	1.210	1.046	1.400	0.074	NA
+Wang, *et al.*	14.363%	1.189	1.039	1.362	0.069	0.012
+Ray, *et al.*	5.420%	1.154	0.988	1.347	0.079	0.071
+Wei, *et al.*	16.394%	1.204	0.987	1.469	0.101	0.067
+Xu, *et al.*	17.795%	1.238	1.061	1.444	0.078	0.007

### Exploration for inconsistency and risk of bias across studies

Differences in the immunohistochemical protocols and techniques used for FOXC1 staining could produce some inconsistencies in FOXC1 detection. Biases that may affect the cumulative evidence include sample selection, because samples were not chosen randomly in all studies, and our inclusion of multivariate cohort studies which, because they evaluate multiple parameters simultaneously, could increase the heterogeneity of our results. We attempted to minimize heterogeneity by limiting study eligibility. We only selected the multivariate cohort design which evaluated FOXC1 expression via immunohistochemical staining. The I^2^ of the meta-analysis study has shown a mild heterogeneity with 24.47% with a *p*-value = 0.258 (Table 3).

**Table 3:** Heterogeneity

tau^2^	Q(df=4)	Heterogeneity *p*-value	*I*^2^
0.008	5.296	0.258	24.472

## DISCUSSION

Accumulating evidence indicates that FOXC1 is involved in tumor development and metastasis. In particular, FOXC1 is a prognostic biomarker for BLBC [24, 26, 27], which is a form of triple-negative breast cancer for estrogen receptor (ER), progesterone receptor (PR), and human epidermal growth factor receptor 2 (HER2). Elevated FOXC1 mRNA expression is associated with a worse overall survival of breast cancer patients [27], as well as with brain and lung metastasis of breast cancer [26, 27]. Therefore, the function of FOXC1 in breast cancer, specifically BLBC, has been extensively investigated. FOXC1 plays a critical role in proliferation, migration, invasion, and epithelial-to-mesenchymal transition (EMT) of breast cancer cells through regulation of EGFR, NF-kB, and MMP7 [28–30], as well as in control of breast cancer stem cell properties by directly interacting with the Gli2 transcription factor [31].

Most of the studies chosen in our meta-analysis suggested that FOXC1 expression and cancer were associated, but none of them reported the relative risk between tumor stage and FOXC1 levels. Our meta-analysis compared the expression of FOXC1 in stage T1-T2 and stage T3-T4 tumors, and we reduced selection bias by restricting our analysis to multivariate cohort studies that detected FOXC1 expression via immunohistochemical staining and based the T-stage definition on tumor size, which does not predict morbidity but is a definitive indicator of tumor growth. Our results indicate that FOXC1 expression is significantly more common in late-stage (T3-T4) tumors than in early stage (T1-T2) tumors and, consequently, that FOXC1 may be a marker for the T-stage of cancer.

## SUPPLEMENTARY MATERIALS TABLE




