# Supplementary material for: Meta-analysis of the likelihood of FOXC1 expression in early- and late-stage tumors
Source: Oncotarget. 2018 Nov 27;9(93):36625–30. doi: 10.18632/oncotarget.26358 (PMC6290959; doi:10.18632/oncotarget.26358)
Supplement: Supplementary file 1 [file oncotarget-09-36625-s001.pdf]

Meta-analysis of the likelihood of FOXC1 expression in early- and late-stage tumors

SUPPLEMENTARY MATERIALS

Supplementary Table 1: FOXC1 and cancer search. See Supplementary_Table_1
