# Supplementary material for: Meta-analysis of the likelihood of FOXC1 expression in early- and late-stage tumors
Source: Oncotarget. 2018 Nov 27;9(93):36625–30. doi: 10.18632/oncotarget.26358 (PMC6290959; doi:10.18632/oncotarget.26358)
Supplement: Supplementary file 2 [file oncotarget-09-36625-s002.docx]

***Supplemental Table for the Midline search results***

| **Pubmed Search results** |
| --- |
| 1. FOXC1 induces cancer stem cell-like properties through upregulation of beta-catenin in NSCLC. |
| 1. The FOXC1/FBP1 signaling axis promotes colorectal cancer proliferation by enhancing the Warburg effect. |
| 1. Combined diagnosis of breast cancer in the early stage by MRI and detection of gene expression. |
| 1. An alternative splicing switch in FLNB promotes the mesenchymal cell state in human breast cancer. |
| 1. The potential pathway of FOXC1 high expression in regulating the proliferation, migration, cell cycle and epithelialmesenchymal transition of basal-like breast cancer and in vivo imaging. |
| 1. FOXC1 plays a crucial role in the growth of pancreatic cancer |
| 1. Targeting EZH2 reactivates a breast cancer subtype-specific anti-metastatic transcriptional program. |
| 1. Forkhead box C1 promotes colorectal cancer metastasis through transactivating ITGA7 and FGFR4 expression. |
| 1. Impact of OGT deregulation on EZH2 target genes FOXA1 and FOXC1 expression in breast cancer cells. |
| 1. CircIRAK3 sponges miR-3607 to facilitate breast cancer metastasis |
| 1. Feasibility of Classification of Triple Negative Breast Cancer by Immunohistochemical Surrogate Markers. |
| 1. System analysis of the regulation of the immune response by CD147 and FOXC1 in cancer cell lines. |
| 1. Research progress on the forkhead box C1. |
| 1. Evaluation of FOXC1 as a therapeutic target for basal-like breast cancer. |
| 1. FOXC1, the new player in the cancer sandbox. |
| 1. FOXC1 in cancer development and therapy: deciphering its emerging and divergent roles. |
| 1. Forkhead box protein C1 promotes cell proliferation and invasion in human cervical cancer. |
| 1. FOXC1-induced non-canonical WNT5A-MMP7 signaling regulates invasiveness in triple-negative breast cancer. |
| 1. Genistein induces apoptosis of colon cancer cells by reversal of epithelial-to-mesenchymal via a Notch1/NF-κB/slug/E-cadherin pathway. |
| 1. Inhibition of lobuloalveolar development by FOXC1 overexpression in the mouse mammary gland. |
| 1. BRCA2 carriers with male breast cancer show elevated tumour methylation. |
| 1. FoxC1 promotes epithelial-mesenchymal transition through PBX1 dependent transactivation of ZEB2 in esophageal cancer. |
| 1. Members of FOX family could be drug targets of cancers. |
| 1. MicroRNA-204-5p inhibits invasion and metastasis of laryngeal squamous cell carcinoma by suppressing forkhead box C1. |
| 1. MicroRNA-374c-5p regulates the invasion and migration of cervical cancer by acting on the Foxc1/snail pathway. |
| 1. FOXC1 Regulates FGFR1 Isoform Switching to Promote Invasion Following TGFβ-Induced EMT. |
| 1. The long noncoding RNA FOXCUT promotes proliferation and migration by targeting FOXC1 in nasopharyngeal carcinoma. |
| 1. Identification of EGF-NF-κB-FOXC1 signaling axis in basal-like breast cancer. |
| 1. miR-582-5p inhibits invasion and migration of salivary adenoid cystic carcinoma cells by targeting FOXC1. |
| 1. FOXC1 overexpression is a marker of poor response to anthracycline-based adjuvant chemotherapy in sporadic triple-negative breast cancer. |
| 1. A New Gene Expression Signature for Triple Negative Breast Cancer Using Frozen Fresh Tissue before Neoadjuvant Chemotherapy. |
| 1. Advances of FOXC1 transcription factor in cancer. |
| 1. Tumor Hypoxia Regulates Forkhead Box C1 to Promote Lung Cancer Progression. |
| 1. FOXC1 promotes proliferation and epithelial-mesenchymal transition in cervical carcinoma through the PI3K-AKT signal pathway. |
| 1. The apoptotic and genomic studies on A549 cell line induced by silver nitrate. |
| 1. Down-regulation of MicroRNA-133 predicts poor overall survival and regulates the growth and invasive abilities in glioma. |
| 1. FOXC1: an emerging marker and therapeutic target for cancer. |
| 1. FOXC1 is associated with estrogen receptor alpha and affects sensitivity of tamoxifen treatment in breast cancer. |
| 1. Single-cell profiling reveals that eRNA accumulation at enhancer-promoter loops is not required to sustain transcription. |
| 1. Cell Cycle M-Phase Genes Are Highly Upregulated in Anaplastic Thyroid Carcinoma. |
| 1. FOXC1 identifies basal-like breast cancer in a hereditary breast cancer cohort. |
| 1. The role and the potential regulatory pathways of high expression of forkhead box C1 in promoting tumor growth and metastasis of basal-like breast cancer. |
| 1. An integrative genomics approach for identifying novel functional consequences of PBRM1 truncated mutations in clear cell renal cell carcinoma (ccRCC). |
| 1. The relationship between nuclear factor (NF)-κB family gene expression and prognosis in triple-negative breast cancer (TNBC) patients receiving adjuvant doxorubicin treatment. |
| 1. FOXC1 promotes melanoma by activating MST1R/PI3K/AKT. |
| 1. Systematic review of peri-operative prognostic biomarkers in pancreatic ductal adenocarcinoma. |
| 1. Screening Driving Transcription Factors in the Processing of Gastric Cancer. |
| 1. Identification of genes associated with renal cell carcinoma using gene expression profiling analysis. |
| 1. FOXC1-induced Gli2 activation: A non-canonical pathway contributing to stemness and anti-Hedgehog resistance in basal-like breast cancer. |
| 1. Tissue-inappropriate derepression of FOXC1 is frequent and functional in human acute myeloid leukemia. |
| 1. Functional Profiling of Human MeCP2 by Automated Data Comparison Analysis and Computerized Expression Pathway Modeling. |
| 1. Erratum to: FOXC1, a target of polycomb, inhibits metastasis of breast cancer cells. |
| 1. miR-133 inhibits pituitary tumor cell migration and invasion via down-regulating FOXC1 expression. |
| 1. FOXC1 is involved in ERα silencing by counteracting GATA3 binding and is implicated in endocrine resistance. |
| 1. Genome-wide association analysis identifies TXNRD2, ATXN2 and FOXC1 as susceptibility loci for primary open-angle glaucoma. |
| 1. Carotenoid Nanovector for Efficient Therapeutic Gene Knockdown of Transcription Factor FOXC1 in Liver Cancer. |
| 1. Yap and Taz play a crucial role in neural crest-derived craniofacial development. |
| 1. miR-4792 inhibits epithelial-mesenchymal transition and invasion in nasopharyngeal carcinoma by targeting FOXC1. |
| 1. FOXC1 Activates Smoothened-Independent Hedgehog Signaling in Basal-like Breast Cancer. |
| 1. Forkhead box C1 induces epithelial‑mesenchymal transition and is a potential therapeutic target in nasopharyngeal carcinoma. |
| 1. Aberrantly Expressed OTX Homeobox Genes Deregulate B-Cell Differentiation in Hodgkin Lymphoma. |
| 1. Frequent Derepression of the Mesenchymal Transcription Factor Gene FOXC1 in Acute Myeloid Leukemia. |
| 1. FoxC1: Novel Regulator of Inflammation-Induced Metastasis in Hepatocellular Carcinoma. |
| 1. MicroRNA-495 downregulates FOXC1 expression to suppress cell growth and migration in endometrial cancer. |
| 1. Interleukin-8 Induces Expression of FOXC1 to Promote Transactivation of CXCR1 and CCL2 in Hepatocellular Carcinoma Cell Lines and Formation of Metastases in Mice. |
| 1. Dual regulation by microRNA-200b-3p and microRNA-200b-5p in the inhibition of epithelial-to-mesenchymal transition in triple-negative breast cancer. |
| 1. Diagnosis of Basal-Like Breast Cancer Using a FOXC1-Based Assay. |
| 1. Alternatively spliced isoforms of WT1 control podocyte-specific gene expression. |
| 1. MicroRNA-138-5p regulates pancreatic cancer cell growth through targeting FOXC1. |
| 1. Jun-regulated genes promote interaction of diffuse large B-cell lymphoma with the microenvironment. |
| 1. Forkhead box C1 promoter upstream transcript, a novel long non-coding RNA, regulates proliferation and migration in basal-like breast cancer. |
| 1. Expression of FOXC1 and its relationship with E-cadherin in nasopharyngeal carcinoma tissues]. |
| 1. DNA methylation status of key cell-cycle regulators such as CDKNA2/p16 and CCNA1 correlates with treatment response to doxorubicin and 5-fluorouracil in locally advanced breast tumors. |
| 1. Genome-wide DNA methylation profiling reveals parity-associated hypermethylation of FOXA1. |
| 1. [Expressions of FOXC1 and MMP-7 in molecular subtypes of breast cancer and their association with clinicopathological characteristics]. |
| 1. miR-639 regulates transforming growth factor beta-induced epithelial-mesenchymal transition in human tongue cancer cells by targeting FOXC1. |
| 1. FOXC1 is a critical mediator of EGFR function in human basal-like breast cancer. |
| 1. Deregulated FOX genes in Hodgkin lymphoma. |
| 1. A novel long non-coding RNA FOXCUT and mRNA FOXC1 pair promote progression and predict poor prognosis in esophageal squamous cell carcinoma. |
| 1. VGLL1 expression is associated with a triple-negative basal-like phenotype in breast cancer. |
| 1. The expression and functional role of a FOXC1 related mRNA-lncRNA pair in oral squamous cell carcinoma. |
| 1. Lmx1b and FoxC combinatorially regulate podocin expression in podocytes. |
| 1. Repressed BMP signaling reactivates NKL homeobox gene MSX1 in a T-ALL subset. |
| 1. Overexpression of FOXC1 correlates with poor prognosis in gastric cancer patients. |
| 1. Oncogenic deregulation of NKL homeobox gene MSX1 in mantle cell lymphoma. |
| 1. Gene expression patterns combined with bioinformatics analysis identify genes associated with cholangiocarcinoma. |
| 1. Quantitative DNA methylation analyses reveal stage dependent DNA methylation and association to clinico-pathological factors in breast tumors. |
| 1. Low trichorhinophalangeal syndrome 1 gene transcript levels in basal-like breast cancer associate with mesenchymal-to-epithelial transition. |
| 1. Spry1 and Spry2 are necessary for eyelid closure. |
| 1. FOXC1 is enriched in the mammary luminal progenitor population, but is not necessary for mouse mammary ductal morphogenesis. |
| 1. Genetic polymorphisms associated with oxaliplatin-induced peripheral neurotoxicity in Japanese patients with colorectal cancer. |
| 1. Copy number aberrations of genes regulating normal thymus development in thymic epithelial tumors. |
| 1. Circulating breast tumor cells exhibit dynamic changes in epithelial and mesenchymal composition. |
| 1. Foxc1 controls the growth of the murine frontal bone rudiment by direct regulation of a Bmp response threshold of Msx2. |
| 1. High expression of FOXC1 is associated with poor clinical outcome in non-small cell lung cancer patients. |
| 1. High level of FOXC1 expression is associated with poor prognosis in pancreatic ductal adenocarcinoma. |
| 1. FOXC1 contributes to microvascular invasion in primary hepatocellular carcinoma via regulating epithelial-mesenchymal transition. |
| 1. Overexpression of forkhead box C1 promotes tumor metastasis and indicates poor prognosis in hepatocellular carcinoma. |
| 1. The forkhead box transcription factor FOXC1 promotes breast cancer invasion by inducing matrix metalloprotease 7 (MMP7) expression. |
| 1. Single cell profiling of circulating tumor cells: transcriptional heterogeneity and diversity from breast cancer cell lines. |
| 1. Implication of TGF-β as a survival factor during tumour development. |
| 1. Apoptosis-, proliferation, immune function-, and drug resistance- related genes in ER positive, HER2 positive and triple negative breast cancer. |
| 1. Raf-1 oncogenic signaling is linked to activation of mesenchymal to epithelial transition pathway in metastatic breast cancer cells. |
| 1. Gene expression and pathologic response to neoadjuvant chemotherapy in breast cancer. |
| 1. FOXC1 regulates the functions of human basal-like breast cancer cells by activating NF-κB signaling. |
| 1. BRCA1 and GATA3 corepress FOXC1 to inhibit the pathogenesis of basal-like breast cancers. |
| 1. Life stage differences in mammary gland gene expression profile in non-human primates. |
| 1. Polymorphic markers associated with severe oxaliplatin-induced, chronic peripheral neuropathy in colon cancer patients. |
| 1. DNA methylation changes in cells regrowing after fractioned ionizing radiation. |
| 1. FOXC1, a target of polycomb, inhibits metastasis of breast cancer cells. |
| 1. Basal-like breast cancer defined by FOXC1 expression offers superior prognostic value: a retrospective immunohistochemical study. |
| 1. Dysregulation of microRNA-204 mediates migration and invasion of endometrial cancer by regulating FOXC1. |
| 1. Epidermal Growth Factor Receptor (EGFR) mutation analysis, gene expression profiling and EGFR protein expression in primary prostate cancer. |
| 1. Autocrine TGF-β protects breast cancer cells from apoptosis through reduction of BH3-only protein, Bim. |
| 1. Core epithelial-to-mesenchymal transition interactome gene-expression signature is associated with claudin-low and metaplastic breast cancer subtypes. |
| 1. FOXC1 is a potential prognostic biomarker with functional significance in basal-like breast cancer. |
| 1. DNA methylation profiling in doxorubicin treated primary locally advanced breast tumours identifies novel genes associated with survival and treatment response. |
| 1. Frequent aberrant DNA methylation of ABCB1, FOXC1, PPP2R2B and PTEN in ductal carcinoma in situ and early invasive breast cancer. |
| 1. Canonical Wnt signaling regulates Foxc1/2 expression in P19 cells. |
| 1. Gene expression of forkhead transcription factors in the normal and diseased human prostate. |
| 1. Cell type-specific DNA methylation patterns in the human breast. |
| 1. Chromosomal abnormality at 6p25.1-25.3 identifies a susceptibility locus for hypothalamic hamartoma associated with epilepsy. |
| 1. Gene expression profiles relate to SS18/SSX fusion type in synovial sarcoma. |
| 1. Tissue inhibitor of metalloproteinases-1 stimulates gene expression in MDA-MB-435 human breast cancer cells by means of its ability to inhibit metalloproteinases. |
| 1. The lacrimal gland transcriptome is an unusually rich source of rare and poorly characterized gene transcripts. |
| 1. Human FOX gene family (Review). |
| 1. Identification of FOXC1 as a TGF-beta1 responsive gene and its involvement in negative regulation of cell growth. |
| 1. The mouse Fkh1/Mf1 gene: cDNA sequence, chromosomal localization and expression in adult tissues. |

| **The Multivariant Cohort Studies** |
| --- |
| 1. Overexpression of Forkhead Box C1 Promotes Tumor Metastasis and Indicates Poor Prognosis in Hepatocellular Carcinoma |
| 1. miR-582-5p inhibits invasion and migration of salivary adenoid cystic carcinoma cells by targeting FOXC1 |
| 1. Basal-Like Breast Cancer Defined by FOXC1 Expression Offers Superior Prognostic Value: A Retrospective Immunohistochemical Study |
| 1. High expression of FOXC1 is associated with poor clinical outcome in non-small cell lung cancer patients |
| 1. Overexpression of FOXC1 correlates with poor prognosis in gastric cancer patients |
| 1. Down-regulation of MicroRNA-133 predicts poor overall survival and regulates the growth and invasive abilities in glioma |
| 1. miR-639 regulates transforming growth factor beta-induced epithelial–mesenchymal transition in human tongue cancer cells by targeting FOXC1 |
| 1. High level of FOXC1 expression is associated with poor prognosis in pancreatic ductal adenocarcinoma |
| 1. Forkhead box C1 promotes colorectal cancer metastasis through transactivating ITGA7 and FGFR4 expression |
| 1. The long noncoding RNA FOXCUT promotes proliferation and migration by targeting FOXC1 in nasopharyngeal carcinoma |
| 1. MicroRNA-204-5p inhibits invasion and metastasis of laryngeal squamous cell carcinoma by suppressing forkhead box C1 |
| 1. Feasibility of Classification of Triple Negative Breast Cancer by Immunohistochemical Surrogate Markers |
| 1. MicroRNA-374c-5p regulates the invasion and migration of cervical cancer by acting on the Foxc1/snail pathway |
| 1. Forkhead box protein C1 promotes cell proliferation and invasion in human cervical cancer |
| 1. A novel long non-coding RNA FOXCUT and mRNA FOXC1 pair promote progression and predict poor prognosis in esophageal squamous cell carcinoma |
| 1. Forkhead box C1 induces epithelial‑mesenchymal transition and is a potential therapeutic target in nasopharyngeal carcinoma |
| 1. Diagnosis of Basal-Like Breast Cancer Using a FOXC1-Based Assay |
| 1. FOXC1 identifies basal-like breast cancer in a hereditary breast cancer cohort |
| 1. The relationship between nuclear factor (NF)-κB family gene expression and prognosis in triplenegative breast cancer (TNBC) patients receiving adjuvant doxorubicin treatment |
| 1. FOXC1 promotes proliferation and epithelial-mesenchymal transition in cervical carcinoma through the PI3K-AKT signal pathway |
| 1. FOXC1 overexpression is a marker of poor response to anthracycline‑based adjuvant chemotherapy in sporadic triple‑negative breast cancer |
| 1. Polymorphic Markers Associated With Severe Oxaliplatin-Induced, Chronic Peripheral Neuropathy in Colon Cancer Patients |

| **The Multivariant Cohort Studies Which Matched Our Inclusion Criteria** |
| --- |
| 1. Overexpression of Forkhead Box C1 Promotes Tumor Metastasis and Indicates Poor Prognosis in Hepatocellular Carcinoma |
| 1. miR-582-5p inhibits invasion and migration of salivary adenoid cystic carcinoma cells by targeting FOXC1 |
| 1. Basal-Like Breast Cancer Defined by FOXC1 Expression Offers Superior Prognostic Value: A Retrospective Immunohistochemical Study |
| 1. High expression of FOXC1 is associated with poor clinical outcome in non-small cell lung cancer patients |
| 1. Overexpression of FOXC1 correlates with poor prognosis in gastric cancer patients |
